# Supplementary material for: A consensus set of genetic vulnerabilities to ATR inhibition
Source: Open Biol. 2019 Sep 11;9(9):190156. doi: 10.1098/rsob.190156 (PMC6769295; doi:10.1098/rsob.190156)
Supplement: Supplemental Figures_v4.pdf [file rsob190156supp1.pdf]

Supplemental Information to the article

A consensus set of genetic vulnerabilities to ATR inhibition

Nicole Hustedt[1,2], Alejandro Álvarez-Quilón[1], Andrea McEwan[1], Jing Yi Yuan[1], Tiffany Cho[1,3], Lisa Koob[1,5], Traver Hart[4], and Daniel Durocher[1,3#]

1 Lunenfeld-Tanenbaum Research Institute, Mount Sinai Hospital, 600 University Avenue, Toronto, ON, M5G 1X5, Canada

2 Present address: Ridgeline Therapeutics, Hochbergerstrasse 60 C, CH-4057 Basel, Switzerland

3 Department of Molecular Genetics, University of Toronto, 1 King's College Circle, Toronto, ON, M5S 1A8, Canada

4 Department of Bioinformatics and Computational Biology, University of Texas MD Anderson Cancer Center, Houston, Texas, USA

5 Present address: Netherlands Cancer Institute, Plesmanlaan 121, 1066 CX Amsterdam, The Netherlands

# corresponding author

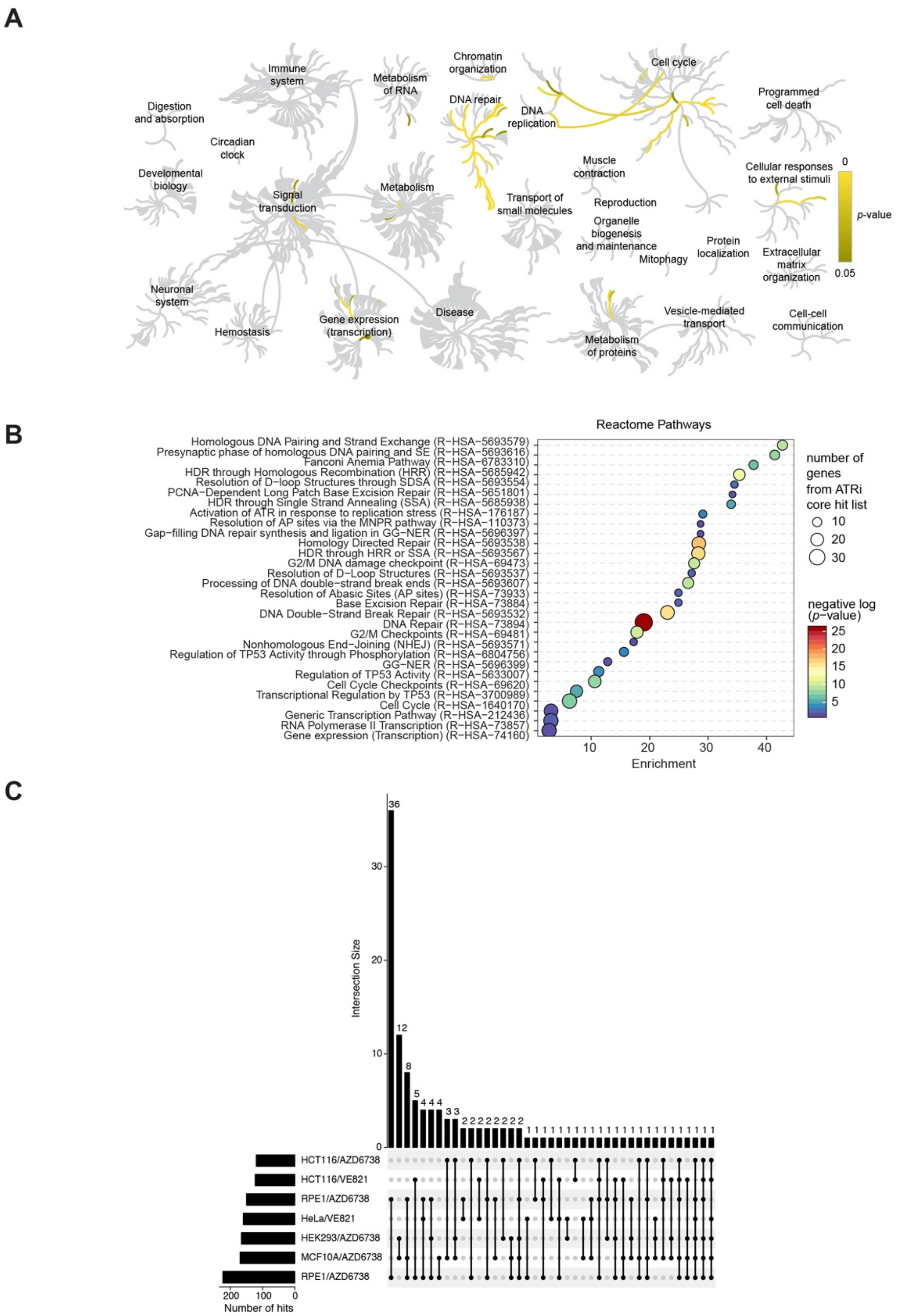

Supplementary Figure 1. Reactome pathways enrichment analysis and screen comparison. a, The117-gene ATRi core gene set was analyzed for enrichment of Reactome pathways (https://reactome.org). Shown is Reactome pathway browser with significantly enriched pathways highlighted in yellow. Color bar indicates *p*-value. b, Results of enrichment analysis of Reactome pathways (http://geneontology.org/page/go-enrichment-analysis) as in Figure 1c and d. Shown are all enriched Reactome pathways except unclassified genes. HDR, homology-directed repair; SDSA, synthesis-dependent strand annealing; MNPR, multiple-nucleotide patch replacement; GG-NER, global genome nucleotide excision repair; HRR, homologous recombination repair, SSA, single strand annealing. c, UpSet plot comparing the overlap between screens.

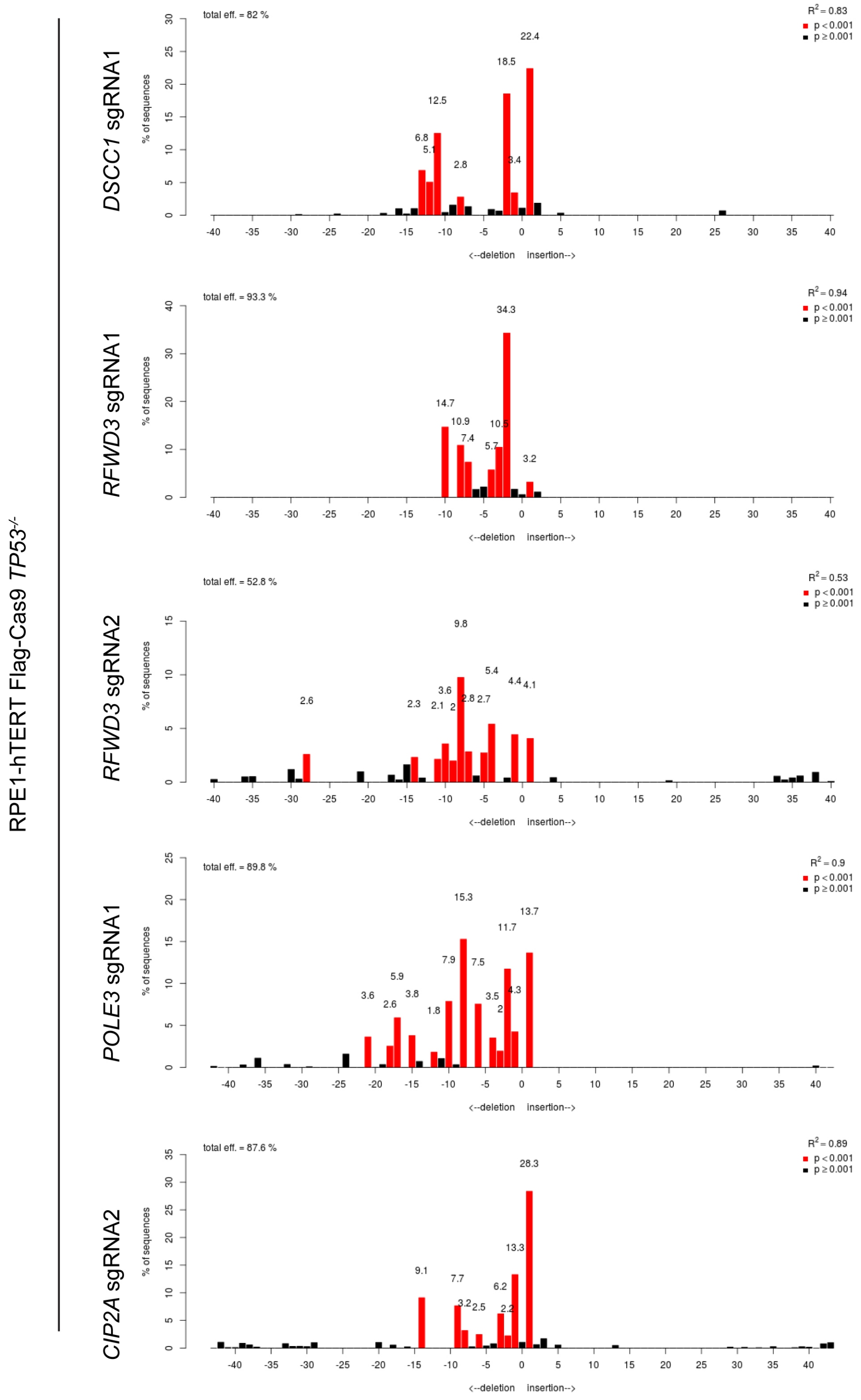

Supplementary Figure 2. Editing efficiency of selected sgRNAs. Genomic DNA was isolated from RPE1-hTERT Flag-Cas9 *TP53*$^{-/-}$ cells transduced with indicated sgRNAs (for sequences see Supplementary Table 3). The region around the sgRNA targeting site was amplified by PCR and sequenced. Results from TIDE analysis of obtained sequences are shown.

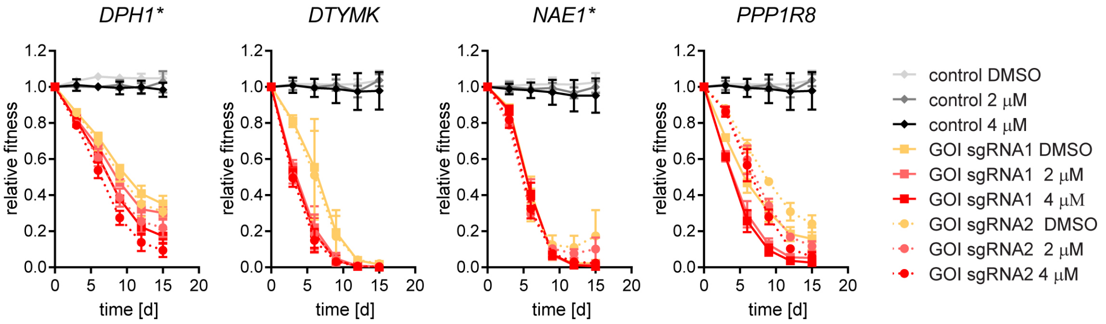

Supplementary Figure 3. Hit validation using two sgRNAs. a, Results of two-color competitive growth assays. RPE1-hTERT Flag-Cas9 *TP53*$^{-/-}$ cells were transduced with either an empty GFP vector (control) or one of two sgRNAs targeting *DPH1*, *DTYMK*, *NAE1* or *PPP1R8* coupled with GFP as well as an sgRNA targeting lacZ coupled with mCherry and treated with indicated concentrations of VE-821. Asterisks indicate genes that are not part of ATRi core gene set. Error bars represent standard deviation of three biologically independent experiments.

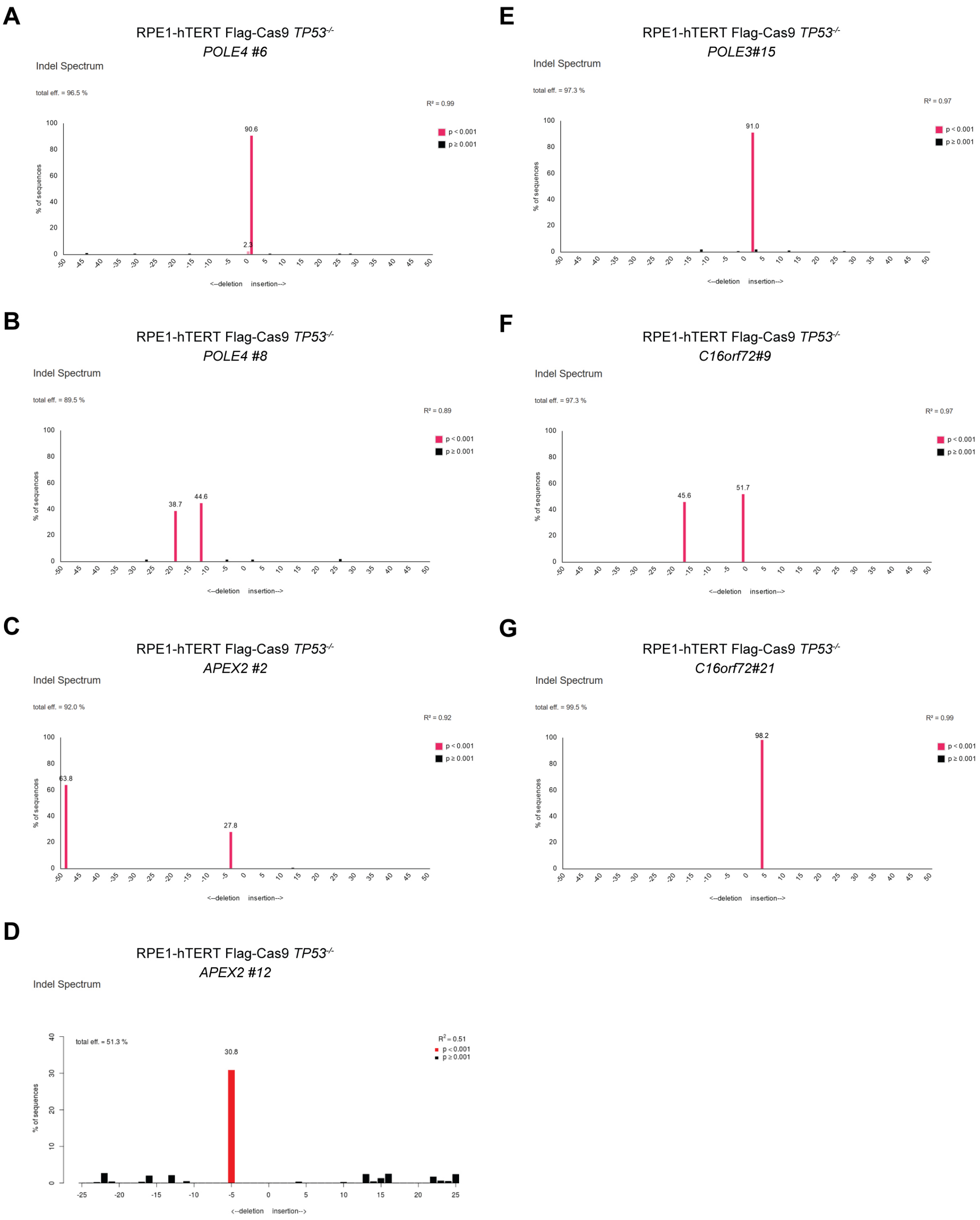

second allele: 149 bp insertion at position +12 of open reading frame

Supplementary Figure 4. Validation of clonal KO cell lines by TIDE analysis. a-g, TIDE analysis of sequences from indicated clones surrounding the targeting sites of sgRNAs used to create gene knockouts.

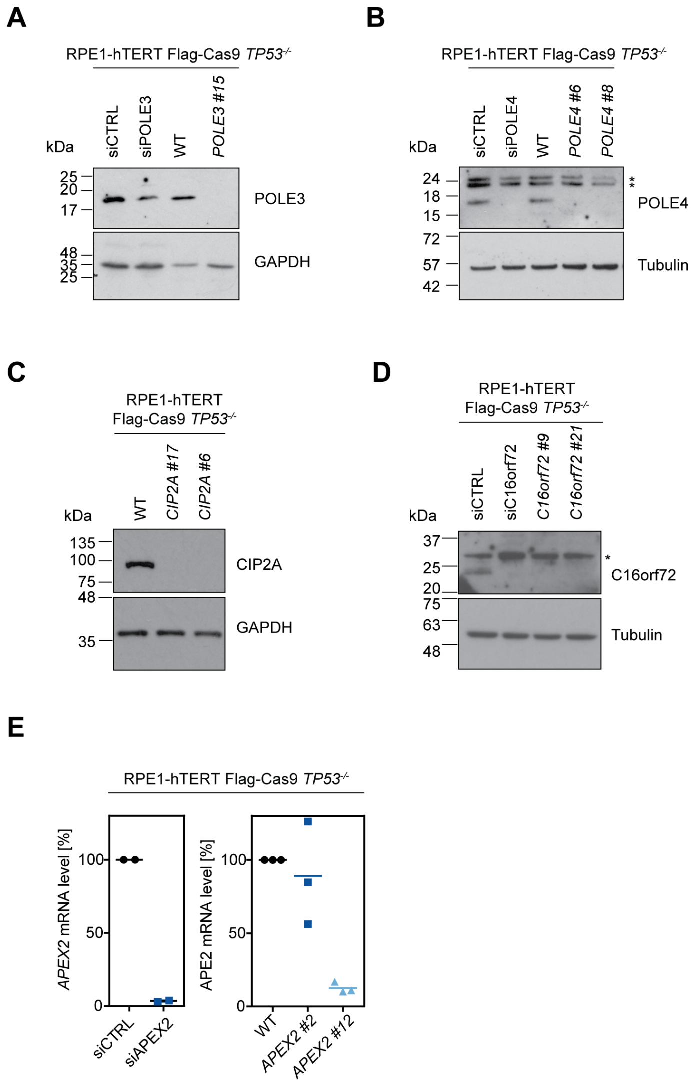

Supplementary Figure 5. Validation of clonal KO cell lines by immunoblotting or qPCR. a-c, Immunoblotting to assess loss of protein expression in clonal knockout cell lines. siRNA-mediated knockdown was used to control for antibody specificity in the case of POLE3 and POLE4 antibodies. Antibodies targeting alpha-tubulin (Tubulin) or GAPDH were used as loading controls. Numbers indicate molecular mass in kDa. Asterisks indicate unspecific bands. d, mRNA level analysis of *APEX2* after siRNA mediated *APEX2* knockdown as assay control and in *APEX2*$^{-/-}$ clones. Clone #2 showed mRNA levels similar to parental (WT) cells but has frameshifting mutations (see panel D) and was sensitive to ATR inhibitor treatment (see Figure 4).

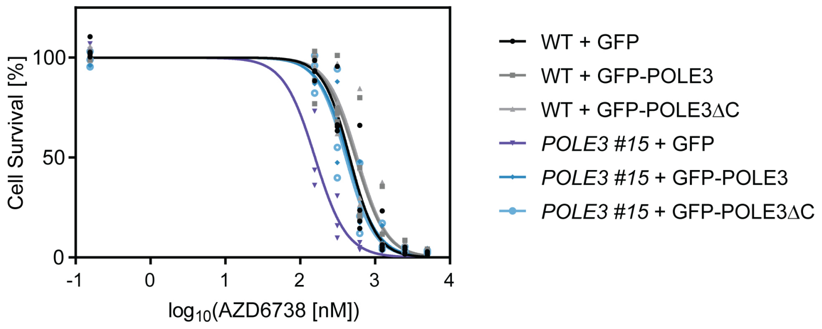

Supplementary Figure 6. POLE3ΔC mutant is not sensitive to ATRi by in an Incucyte assay. Cell survival of RPE1-hTERT Flag-Cas9 *TP53*$^{-/-}$ (WT) or the indicated *POLE3*$^{-/-}$ clone expressing GFP, GFP-POLE3 or GFP-POLE3ΔC treated with indicated concentrations of ATR inhibitor (AZD6738) was determined by monitoring growth in an Incucyte instrument. Data are from three biologically independent experiments.
